# Harnessing mRNA for the expression of monoclonal IgG and IgA in non-human primates

**DOI:** 10.3389/fimmu.2025.1700041

**Published:** 2026-01-16

**Authors:** Romy Rouzeau, Hayden R. Schmidt, Cailin E. Deal, Joel D. Allen, Dawn M. Dudley, Iszac Burton, Eva G. Rakasz, Obadiah Plante, Max Crispin, Andrea Carfi, Devin Sok

**Affiliations:** 1 IAVI Neutralizing Antibody Center, San Diego, CA, United States; 2 Moderna, Inc., Cambridge, MA, United States; 3 School of Biological Sciences, University of Southampton, Southampton, United Kingdom; 4 Wisconsin National Primate Research Center, University of Wisconsin-Madison, Madison, WI, United States; 5 Department of Immunology and Microbiology, The Scripps Research Institute, La Jolla, CA, United States; 6 Consortium for HIV/AIDS Vaccine Development (CHAVD), The Scripps Research Institute, La Jolla, CA, United States

**Keywords:** human immunodeficiency virus (HIV), messenger ribonucleic acid lipid nanoparticles (mRNA-LNP), monoclonal antibody (mAb), non-human primate (NHP), pharmacokinetics (PK)

## Abstract

**Background:**

Monoclonal antibodies (mAbs) are an increasingly essential class of medicines across many disease areas. In the human body, there are five antibody isotypes, each with potential therapeutic benefits for different disease indications. However, 97% of all clinically approved mAbs are produced as the IgG isotype, largely due to challenges associated with recombinantly producing non-IgG isotypes like IgM or IgA, which have additional N-linked glycan sites and can present as multivalent oligomers. One potential way to circumvent this challenge is to express mAbs in situ using mRNA encapsulated in lipid nanoparticles (LNP), bypassing the need for recombinant protein production.

**Objective:**

Here, we demonstrate the feasibility of expressing a mAb as both IgG and IgA in non-human primates (NHPs) using mRNA-LNPs.

**Methods:**

We express ePGDM1400v9, a broadly neutralizing mAb targeting human immunodeficiency virus (HIV), in both IgG1 and IgA2 formats by infusing NHPs with LNPs containing the appropriate mRNAs.

**Results:**

Though IgA2 expression levels were low, both formats were detectable in serum within one day of LNP infusion in all NHPs, and both were detectable in mucosal secretions of most animals. Importantly, serum mRNA-produced IgG1 and IgA2 retained HIV-neutralizing function. Furthermore, mass spectrometry analysis confirmed that mAbs of either isotype produced *in situ* exhibited glycosylation patterns highly similar to that of native antibody, which is likely to confer therapeutic advantages.

**Conclusion:**

Altogether, this work demonstrates that mRNA-LNPs can be used to express native like mAbs of non-IgG isotypes in primates at detectable levels and enables further development and optimization of non-IgG mAb constructs.

## Introduction

Monoclonal antibodies (mAbs) are an essential class of therapeutics for indications across cancer, neurological disease, autoimmune disorders, and infectious disease (1). These biologics hold several advantages over traditional small molecule drugs, including their target specificity and versatility, excellent safety profile, amenability to engineering, and (as demonstrated during the SARS-CoV-2 pandemic) the rapid speed with which they can be identified and developed. These properties have made mAbs one of the fastest growing classes of medicines over the decade (1).

In nature, there are five main isotypes of antibodies that differ in their constant domains, all exhibiting distinct immunological effector functions and recruitment of different immune effector cells. Despite this diversity, almost all immunoglobulins (Ig) that have been approved or are pending approval are of the IgG isotype (97%) due to the ease of manufacturing and favorable pharmacokinetic properties (2) (the non-IgG molecules listed being scFv, Fab, or single-domain V_HH_). As such, less research and effort has been made on optimizing production and purification of alternative isotypes such as IgM and IgA, even when these isotypes may provide disease-specific therapeutic advantages over IgGs.

A potential solution to these challenges is to express mAbs *in situ* using lipid nanoparticle (LNP)-encapsulated messenger ribonucleic acid (mRNA) (3). As a platform, mRNA inherently bypasses the need for protein expression and purification (4), thereby enabling characterization and development of molecules such as IgA, which has been largely untapped because of manufacturing difficulties. The chemistry and manufacturing of mRNA remain the same, regardless of protein, thus allowing for rapid production of different molecules. An additional advantage over recombinantly produced mAbs is the potential for more native-like glycosylation patterns when produced *in situ* from mRNA (5). This has proven to be advantageous for complex molecules like IgA as a lack of sufficient sialylation, which is reduced in common antibody production cell lines, can result in rapid clearance *in vivo* (6). Nonetheless, IgA mAbs could be transformative therapeutics against mucosal pathogens due to their privileged mucosal access as IgA dimers, complexed together with the J chain (7, 8), which enables transcytosis via the polymeric immunoglobulin receptor (pIgR).

To date, mRNA-delivered mAbs against human immunodeficiency virus (HIV) (9, 10), tumors (11, 12), botulin toxin (12), rabies virus (12), respiratory syncytial virus (13), and Chikungunya virus (14) have been shown to be protective in mice. The expression of mRNA-encoded anti-Chikungunya virus mAb (mRNA-1944) reached theoretical protection levels in both non-human primates (NHP) (12) and healthy adults in a Phase I clinical trial (15). The above mAbs represent various formats and constructs, though all are expressed as IgGs or in formats that have been validated in the clinic using recombinant protein products. More recently, we reported the successful expression of IgA mAbs in mice, which were able to prevent mucosal colonization of pathogenic *Salmonella* and *Pseudomonas* (5).

Here, we build upon this work by demonstrating that ePGDM1400v9, the enhanced version of a broadly neutralizing antibody (bnAb) against HIV (16), can be expressed in NHPs as either a human IgG1 or a human IgA2 mAb. We show that mRNA-expressed ePGDM1400v9 IgG1, containing an “LS” half-life extension mutation (17), persist in serum for time periods comparable to its passively administered recombinant isotype. In comparison, mRNA-delivered IgA2 exhibited log-shorter half-life, but both mRNA-delivered IgG and IgA isotypes neutralized HIV pseudovirus *in vitro*.

Altogether, this work demonstrates that mAbs can be produced *in situ* in primates via mRNA-LNPs as either IgG or IgA isotypes, and acquire native-like glycosylation while retaining the same functional activities as their recombinant counterparts.

## Results

### Generation and validation of antibodies and assays

To facilitate the accurate measurement of ePGDM1400v9 in NHP tissues and serum, we acquired anti-idiotype antibodies specific to the variable domain of ePGDM1400v9. The anti-idiotype antibody used in this work, aPGDM01, was produced recombinantly as a mouse IgG2a and was shown to bind to recombinant ePGDM1400v9 as a Fab, human IgG1, human IgG2, monomeric human IgA2 and dimeric human IgA2 with high affinity and specificity by surface plasmon resonance (**Supplementary Figure S1A**). Importantly, though recombinant ePGDM1400v9 IgA2 was purified as a mixture of monomers and dimers (**Supplementary Figures S1B, C**), ELISAs using purified dimer or purified monomer confirmed that aPGDM01 binding was not influenced by ePGDM1400v9 IgA2’s oligomeric state (**Supplementary Figure S1D**). To measure ePGDM1400v9 concentrations in NHP sera, an electrochemiluminescence (ECL) assay was developed. This assay was able to accurately measure recombinant ePGDM1400v9 spiked into NHP serum for both IgG1 (**Supplementary Figure S1E**) and IgA2 (**Supplementary Figure S1F**) formats.

### mRNA-derived ePGDM1400v9 is produced *in vivo* and is detectable in serum and mucosal secretions

**Figure 1:**
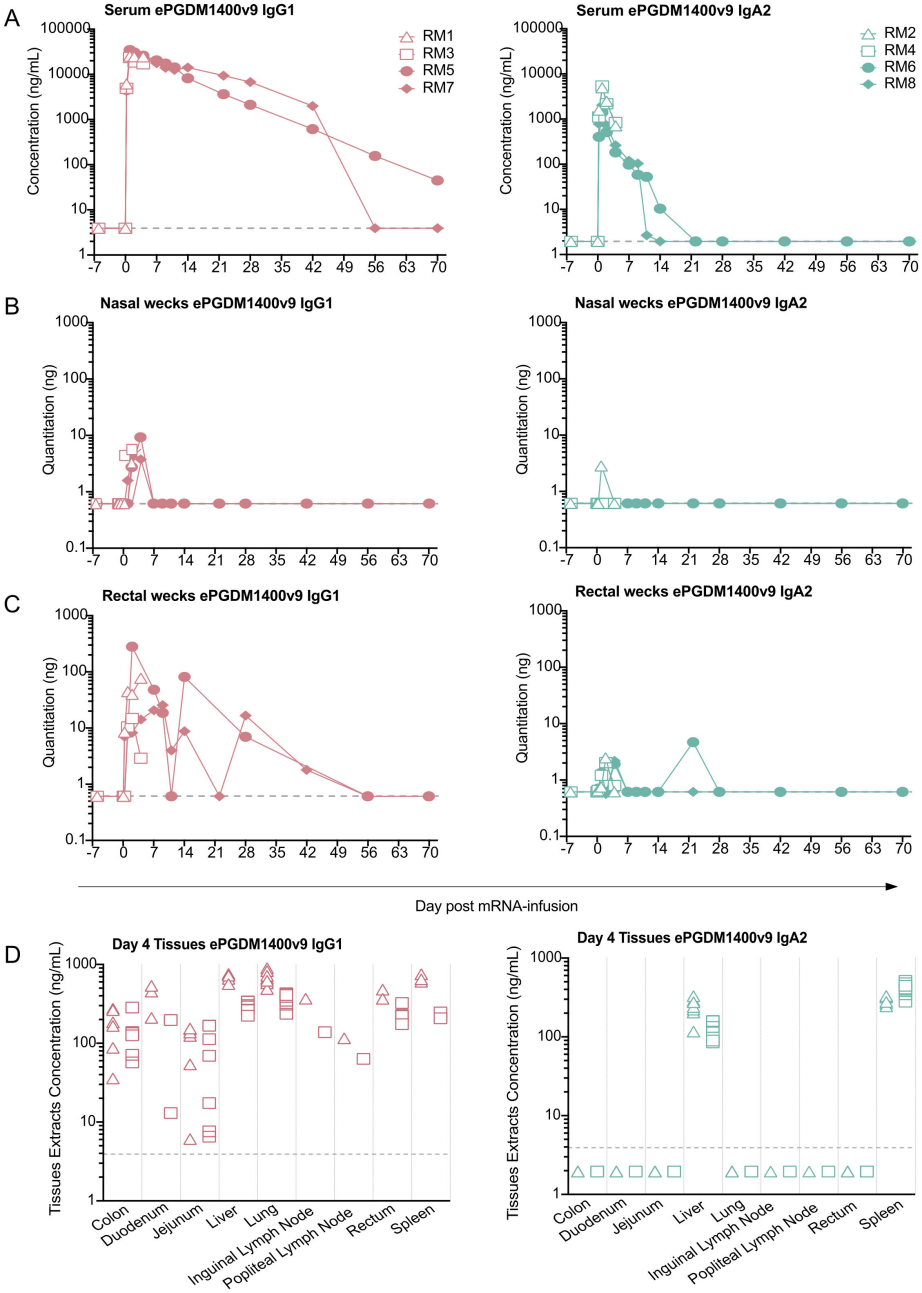
mRNA-mediated expression ePGDM1400v9 IgG1 and IgA2 in NHP serum and tissues. Longitudinal quantification of ePGDM1400v9 IgG1 or IgA2 in mRNA-transfused macaques in: sera **(A)** by ECL, nasal **(B)** and rectal **(C)** wecks by ELISA. The nasal weck sample from IgA2-receiver RM2 collected at d2 post-transfusion was excluded due to the presence of blood on the weck. Similarly, rectal weck samples from IgG1-receiver RM5 collected d1, d4, d21 and d42 post-transfusion, and the sample from IgG1-receiver RM7 collected at d70 post-transfusion, were excluded from analysis due to the presence of blood on the weck. Quantification of ePGDM1400v9 IgG1 or IgA2 **(D)** in tissue in necropsied animals at day 4 post-transfusion by ECLIA. Note that tissues were not perfused at necropsy, so the measurements here represent the total antibody in each tissue and the associated vasculature. The dotted line represents the limit of detection.

We sought to determine the kinetics and quantity of expression of mRNA expressed ePGDM1400v9 as an IgG1 and an IgA2 (**Figure 1**). Two groups of 4 rhesus macaques (8 total) received a 1 mg/kg intravenous transfusion of mRNA-LNPs encoding for either ePGDM1400v9 human IgG1-LS or ePGDM1400v9 IgA2 (**Supplementary Figure S2**). IgA2 was chosen over IgA1 for two reasons: 1) Our previous work on mRNA-mediated IgA expression in mice successfully used this isotype (5), 2) IgA2 has a shorter hinge and is thus less likely to undergo bacterial protease cleavage (18). On day 4 post-transfusion, two animals from each group were necropsied, and select tissues were harvested for biodistribution analysis. The remaining animals were necropsied on day 70 post-transfusion and the same tissues were harvested. No adverse events were observed in any of the animals during the study.

All animals of both groups exhibited detectable levels of ePGDM1400v9 in sera that persisted up to 4 d post-transfusion, when two animals from each group were necropsied (RM1 and RM3 for the IgG receivers, and RM2 and RM4 for the IgA receivers, **Figure 1A**, **Supplementary Table S1**). Across all animals, serum levels of both IgG1 and IgA2 ePGDM1400v9 peaked at 1-day post-transfusion, though IgG levels were significantly higher. Among the four NHPs in each group, peak ePGDM1400v9 IgG1 serum levels ranged from 23.7 – 34.5 µg/mL, while peak ePGDM1400v9 IgA2 serum levels ranged from 1.4 – 5.4 µg/mL (**Supplementary Table S1**). Of the two remaining NHPs that received the IgG1 mAb (RM5 and RM7), one exhibited antibody over the limit of detection (LOD) up to 70 d, while the other dropped below the LOD between 42 d and 56 d. Of the two remaining IgA2-receiving NHPs (RM6 and RM8), one dropped below the LOD between 10 d and 14 d, and the other between 14 d and 21 d.

The serum half-life of ePGDM1400v9 was calculated for each animal not necropsied at 4 d post-infusion. Systemically administered mAbs typically exhibit biphasic decay kinetics, with a rapid distribution phase followed by a slower clearance phase (19), and we found this could accurately model half-life for the mRNA-derived ePGDM1400v9 (**Supplementary Figure S3**, **Supplementary Table S2**). In the two NHPs that received ePGDM1400v9 IgG1 mRNA, RM5 and RM7 exhibited half-lives of 7.3 d and 15.4 d (**Supplementary Table S2**, **Supplementary Figure S3**). For NHPs expressing ePGDM1400v9 as an IgA2, RM6 and RM8 exhibited half-lives of 3.4 d and 3.6 d, which is in the reported range of recombinantly produced monomeric IgA in rhesus macaques (20).

We also measured total ePGDM1400v9 in rectal (**Figure 1B**) and nasal (**Figure 1C**) wecks by ELISA to measure mRNA-derived antibody at mucosal surfaces. In rectal wecks, ePGDM1400v9 IgG1 was detectable in all animals at low levels up to 4 d post-transfusion, while ePGDM1400v9 IgA2 was detectable in most but not all animals over the same period. No ePGDM1400v9 was detectable in any rectal swabs after 4 d post-transfusion. The amount of ePGDM1400v9 IgG1 detected in nasal swabs was much lower than in rectal wecks, and while all animals exhibited detectable antibodies up to 4 d post-transfusion, not all animals had detectable antibody at all timepoints. IgA2 was only detected from one nasal weck from one animal at 1 d post-transfusion. Note that for both rectal and nasal wecks, timepoints were excluded for some animals if blood was observed on the weck (see figure legend for details). Altogether, these data suggest that a small proportion of mRNA-derived ePGDM1400v9 IgG1 and IgA2 did reach mucosal surfaces. Expressing the mAb as an IgA2 did not confer any detectable benefit with respect to mucosal trafficking, though this may be due to limitations in detection methods used and experimental design (see discussion).

### Biodistribution of mRNA-derived ePGDM1400v9 four days post-transfusion

We also measured mRNA-delivered ePGDM1400v9 in select tissues from animals sacrificed at 4 d post-transfusion (two animals per group). Animals were sacrificed at 4 d post-infusion, because 1) peak mAb expression was anticipated to be between 24 and 72 h post-infusion, so 4 d post-infusion would ensure peak expression was reached and that mRNA-derived mAb would have at least 24 h to accumulate in diverse tissues, and 2) studies with recombinant IgG indicate that maximal distribution in relevant tissues (vaginal/rectal) takes approximately 5 days post-administration (21). When expressed as an IgG1, ePGDM1400v9 was detectable in all tissues tested (**Figure 1D**, **Supplementary Table S1**). However, when expressed as an IgA2, the mAb was only detectable in the liver and spleen, where mRNA-LNPs are known to accumulate and express following IV administration (22). These data suggest that while expression levels achieved for the IgG1 construct were high enough to achieve systemic distribution, the IgA2 construct was not detectable in most tissues at 4 d post-transfusion. Note that the tissues examined were not perfused, so ePGDM1400v9 levels measured here represent the total amount of antibody in both the tissue itself and the associated vasculature.

### ePGDM1400v9 IgG and IgA from transfused macaques neutralizes HIV pseudovirus *in vitro*

**Figure 2:**
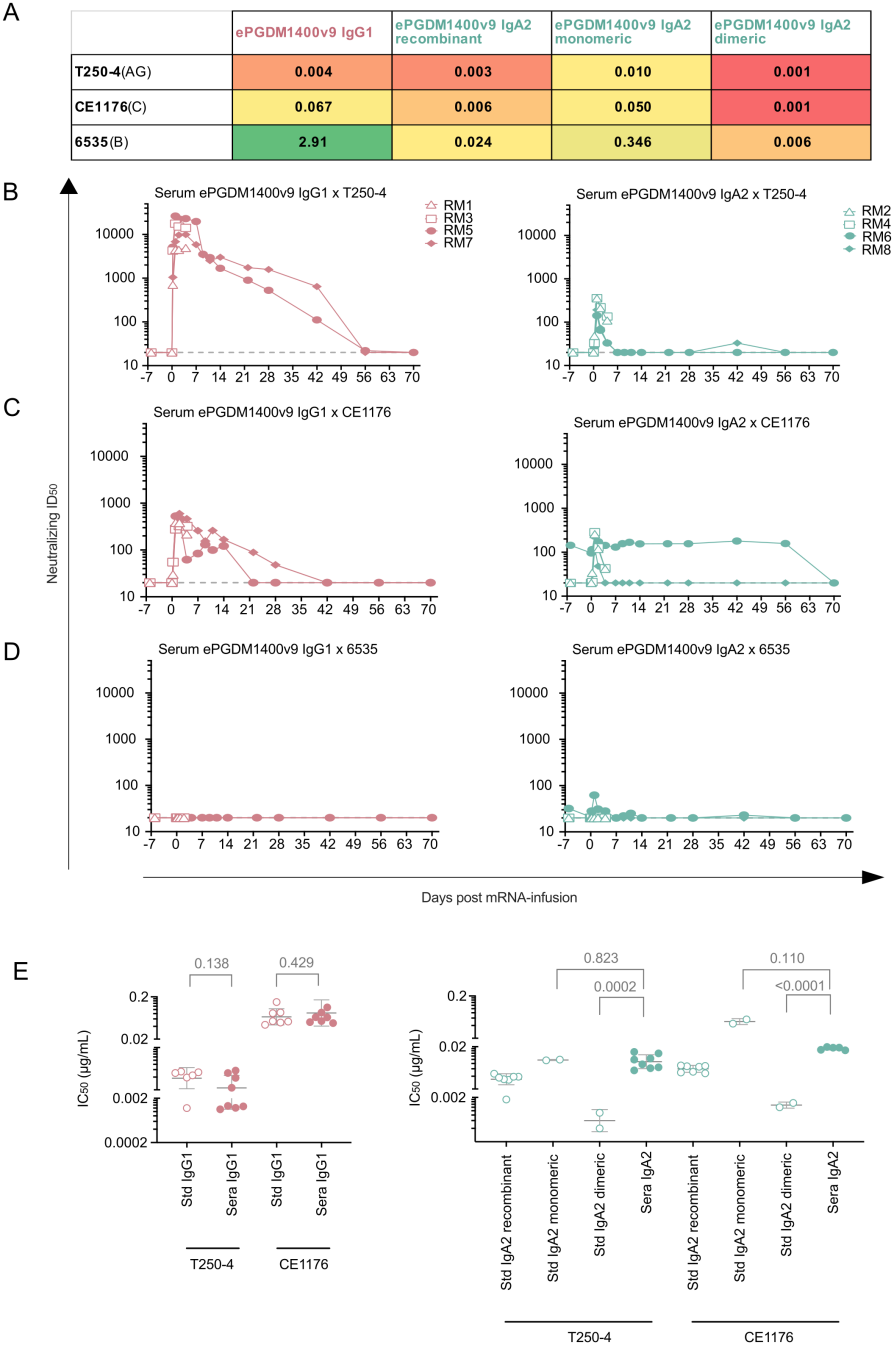
mRNA-derived ePGDM1400v9 IgG1 and IgA2 from NHP sera neutralizing activity as assessed by TZM-bl assay. **(A)***in vitro* IC_50_ of the 3 cross-clade pseudoviruses that were selected based on their low (6535), mid (CE1176) or high (T250-4) neutralization by ePGDM1400v9 IgG1 (in µg/mL). Cold colors (leaning towards green) refer to low potency (high IC_50_) while warm colors (leaning toward red) refer to high potency (low IC_50_. **(B–D)** Neutralization ID_50_ of IgG1 and IgA2 infused sera at each collected timepoint against T250-4 **(B)**, CE1176 **(C)** and 6535 **(D)**. The dotted line represents the limit of detection. **(E)** mRNA-derived ePGDM1400v9 IgG1 (left) and IgA2 (right) IC_50_ against T250–4 and CE1176 at day 1 and 4 post mRNA infusion, extrapolated based on the neutralization ID_50_ and the serum concentration evaluated **(B, C)**, and aligned with control isotype IC_50_ for each of the 3 pseudoviruses. The statistical test was an unpaired and parametric t test with Welch’s correction performed by GraphPad Prism 10.4.2.

We next sought to verify that the ePGDM1400v9 mAbs produced in NHPs by mRNA retained expected neutralization activity against sensitive HIV strains. Both plasma and sera from transfused animals were screened against a panel of three different HIV pseudoviruses, each with a different degree of sensitivity to ePGDM1400v9 IgG1 (**Figure 2A**). Consistent with the observed expression of antibody in all animals, sera were able to selectively neutralize sensitive pseudoviruses (**Figures 2B–D**; **Supplementary Table S3**). No neutralization was observed against the negative control MLV, at any time point. One animal – the IgA2 recipient RM6 – showed pre-existing and nonspecific neutralizing activity against CE1176 (**Supplementary Table S3**), which is probably a consequence of this animal’s involvement in previous studies involving HIV bnAbs (based on internal facility records). As a result, RM6 was removed from the following analyses. Importantly, the serum ID_50_ for each sample correlated well with the serum ePGDM1400v9 concentration measured by ECLIA (**Figure 2D**).

We used the ePGDM1400v9 serum concentrations determined by ECLIA and the neutralization assay ID_50_s to calculate estimated IC_50_ values for each group against each virus at day 1 and 4 timepoints, which facilitated a direct comparison to recombinant ePGDM1400v9 IgG1 (**Figure 2E**). We observed that the potency of the ePGDM1400v9 IgG1 against sensitive pseudoviruses (T250–4 and CE1176) was statistically indistinguishable from its recombinant counterpart, suggesting that the IgG1s produced *in situ* by mRNA had similar neutralization activity to the recombinantly produced IgG1s.

Because the recombinant IgA2 (produced via transient transfection of ePGDM1400v9’s light chain (kappa), heavy chain (IgA2), and j-chain that is required for dimerization) result in a mixture of monomer and dimer (**Supplementary Figures S1B-D**), recombinant monomeric and dimeric species were separated using size exclusion chromatography before comparing the estimated IC_50_ neutralization potency of the sera to recombinant mixed, monomeric, and dimeric species (**Figure 2E**). We observed that dimeric ePGDM1400v9 IgA2 was more potent than the monomeric form, and that the serum IgA2 was generally more similar to the monomeric species in potency, which is consistent with expectations of mostly monomeric IgA2 in serum.

### Glycosylation analysis of mRNA-derived ePGDM1400v9 IgG1

N-linked glycosylation plays a key role in modulating Ig effector functions and is heavily dependent on the producer cell from which the antibodies are derived. As mRNA derived antibodies are produced *in situ*, the factors that influence glycosylation such as metabolite availability as well as the expression level and type of glycosyltransferases, will differ between typical CHO cells used to produce therapeutic Ig and the various cell types that take up the mRNA *in vivo*. Human IgA2 contains 4 potential N-linked glycosylation sites defined as an amino acid sequon consisting of NxS/T, where x is any amino acid except proline. Additionally, IgG contains one N-linked glycosylation site within the Fc. As such, we applied a liquid-chromatography- mass spectrometry (LC-MS) based approach to determine the site-specific glycosylation of ePGDM1400v9 IgA and IgG delivered as mRNA and produced by the macaque.

**Figure 3:**
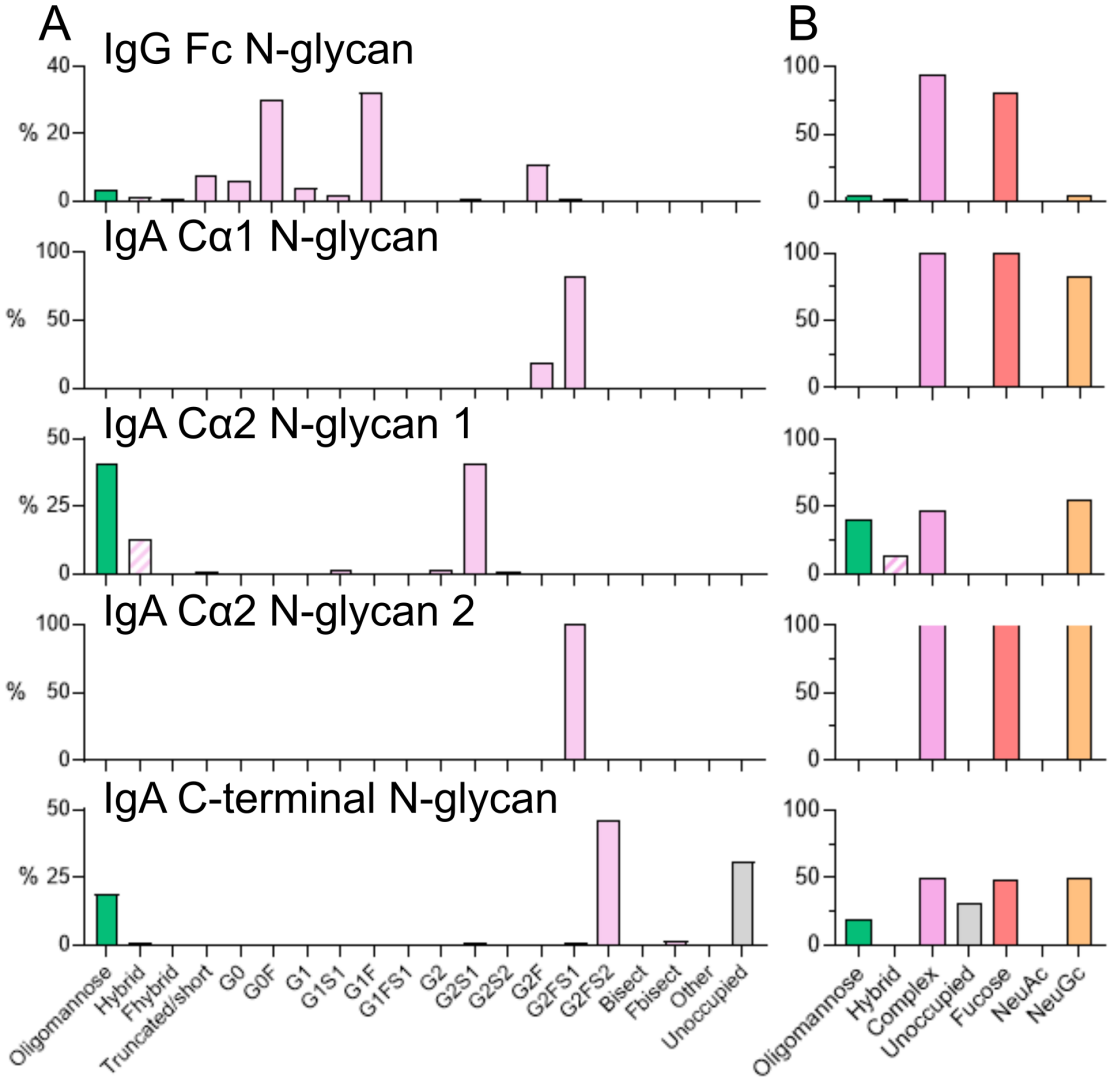
Site-specific glycan analysis of mRNA-derived ePGDM1400v9 IgG and IgA from transfused macaques. **(A)** Summary of the site-specific glycosylation of mRNA-derived ePGDM1400v9 IgA2. The relative proportion of different glycan types is summarized. The total proportion of glycans with compositions corresponding to oligomannose-type glycans is shown in green, hybrid-type glycans in hashed pink, complex-type glycans in pink. The proportion of potential N-linked glycan sites lacking glycan attachment (unoccupied) is shown in grey. As macaques contain NeuGc and no NeuAc was detected, categories denoted by “S” describe glycans containing NeuGc. **(B)** Summarized glycan categories for oligomannose-hybrid- and complex-type glycans. Additionally, the proportion of glycans containing one or more fucose monosaccharides is colored red, NeuAc (sialic acid) in purple and NeuGc (N-glycolylneuraminic acid) in orange. Glycan underoccupancy is also shown in grey.

Analysis of mRNA delivered IgG demonstrated glycan processing that is typical for IgG. The three most abundant glycan compositions detected are within G0F (30%), G1F (32%) and G2F (11%) (**Figure 3A**, **Supplementary Table S5**). The overall fucosylation was high, but not complete, with 81% of detected glycans containing fucose. In contrast to human-derived IgG, no N-acetylneuraminic acid was observed and instead 4% of glycans were modified with N-glycolylneuraminic acid (NeuGc) (**Figure 3B**). This monosaccharide is not found in humans as they lack the CMAH gene (which encodes cytidine monophosphate-N acetylneuraminic acid hydroxylase) (23), but is present naturally in host macaque glycosylation. This enzyme is responsible for converting Neu5Ac (N-acetylneuraminic acid) to NeuGc. A low level of under processed oligomannose-type glycans were also detected, which is also typical for IgG produced in human cell lines (24).

The detected N-linked glycan compositions of IgA2 varied at the site-specific level, displaying a range of processing states including oligomannose-, hybrid- and complex-type glycans. Complex-type glycans were observed across all sites, ranging from 100% abundance on the Cα1 N-glycan site and Cα2 N-glycan site 2 to ~50% on the Cα2 N-glycan site 1 (**Figure 3**, **Supplementary Table S4**). The majority of complex-type N-linked glycans were fucosylated and modified by NeuGc. Minimal levels of NeuAc were observed across all sites. N-linked glycans consisting of HexNAc(4)x were the predominant complex-type glycans observed across all sites, except for Cα2 N-glycan site 1 (**Supplementary File** - Glycopeptide analysis). While linkage analysis was not possible with the selected LC-MS approach, N-linked glycans consisting of 4 N-acetylhexosamines typically correspond to biantennary glycans which comprise the G0, G1 and G2 categories typically used in IgG glycan nomenclature. Under-processed oligomannose-type glycans were detected at two sites, Ca2 glycan site 1 and the N-linked glycan site at the C-terminus. The presence of these glycans has been shown to enhance clearance. Previous analysis of IgA2 delivered mRNA to mice demonstrates a conservation of oligomannose-type glycans across species (5). Finally, potential N-linked glycosylation sites lacking N-linked glycan attachment were observed at the C-terminal N-linked glycosylation site. As glycosylation occurs co-translationally, with the N-glycosylation attachment enzymes co-localizing with the ER-translocon, once translation is complete, N-linked glycosylation sites close to the C-terminus are often skipped as the polypeptide detaches from the ribosome complex (25). Altogether, the glycosylation pattern observed for the mRNA-derived ePGDM1499v9 IgA2 here was similar to that observed for a different IgA2 monoclonal recently expressed by mRNA in mice (5).

## Discussion

We demonstrate the expression of ePGDM1400v9 in NHPs via mRNA as both an IgG1 and an IgA2. The mAb was expressed to detectable levels in the serum of all animals, though IgA2 expression levels were lower than those of IgG1. Both isotypes retained their ability to neutralize sensitive HIV pseudoviruses *in vitro*. Additionally, glycosylation patterns of mAbs produced by mRNA (both IgG1 and IgA2) were more similar to native glycosylation patterns based on the observation of monosaccharide additions that are not usually seen in CHO/HEK-derived material (**Figure 3**). This study presents foundational data of proof-of-concept IgA expression in NHPs that could be further improved with subsequent designs and approaches.

All four NHPs that received mRNA-LNPs coding for ePGDM1400v9 IgA2 expressed detectable levels of the mAb, with peak serum levels of 1.4 – 5.4 µg/mL at 1 d post-transfusion, which was much lower than those observed for the IgG1 construct (**Figure 1A**). There are multiple potential explanations for this low expression level, which are not mutually exclusive. First, the IgA2 construct is transfected as three separate mRNA chains, each coding for a different polypeptide chain: the ePGDM1400v9 IgA heavy chain, the ePGDM1400v9 light chain, and a J-chain to facilitate end-to-end dimerization of IgA2 monomers. Compared to the IgG1 construct, the extra polypeptide in this mix (J-chain) inherently reduces the molar quantity of heavy and light chain delivered per mg of mRNA-LNP. In conjunction with the heavy glycosylation typically present on IgA (26), this significantly increases the molecular complexity of the final product, which could be expected to reduce yields. The mRNA was formulated to provide a molar ratio of 1:2:1 and a mass ratio of 4:4:1 heavy:light:J chains because this ratio enabled the successful expression of IgA2 in mice (5), but this may not be the optimal ratio for producing IgA in NHPs. Additionally, ePGDM1400v9 may be a poor candidate for expression as an IgA yields of the recombinant IgA2 construct in Expi293F cells were substantially lower than the IgG1 construct, and in PBS the IgA2 construct could not be concentrated above 0.22 mg/mL without precipitating whereas the IgG1 construct can be concentrated well over 50 mg/mL. Thus, attempting the experiment with antibodies more amenable to expression as an IgA and optimizing the molar ratio of IgA mRNA chains will likely improve *in situ* IgA expression levels in NHPs.

Two of the most attractive expected advantages of an IgA mAb over traditional IgG mAbs is the enhanced mucosal delivery, and the engagement of FcRαI (25–27). Unfortunately, the low expression levels of ePGDM1400v9 IgA2 observed preclude assessment of either of these features. Neutralization IC_50_ estimates suggest that, as expected, the mRNA-derived IgA2 in the serum and plasma was primarily monomeric (**Figure 2E**), which is not anticipated to traffic to mucosal sites. This is also consistent with the half-lives observed in NHPs receiving ePGDM1400v9 mRNA, which is similar to that of other monomeric IgA mAbs in NHPs (19). At 4 d post-transfusion, we were only able to detect ePGDM1400v9 IgA2 in the spleen and liver, where the mRNA-LNPs are expected to drive expression. However, our serum concentration measurements suggest that by 4 d post-transfusion, serum ePGDM1400v9 IgA2 levels were probably only about 10% of peak levels, which were reached at 1 d post-transfusion (**Figure 1A**). Given that peak levels of mRNA-derived IgA2 were so low, and that a minority of total IgA2 produced may have been dimerized, the expected amount of IgA2 dimer in mucosal tissues at 4 d post-transfusion would likely be below our detection threshold even if it was successfully produced. Similarly, though we do detect low levels of IgA2 in rectal and nasal wecks in some animals during the first few days post-infusion, the low expression level and inherent variability of rectal and nasal weck samples preclude determination of whether the ePGDM1400v9 IgA2 in the nasal or rectal samples was monomeric or dimeric. Therefore, we are currently unable to distinguish between a failure to produce and traffic mRNA-derived IgA2 dimer to the mucosa and a failure to measure the low levels of dimer that were trafficked. Similarly, low expression levels mean that all available mRNA-derived IgA2 mAb was consumed for neutralization and glycosylation analysis and we were unable to assess IgA-mediated effector functions. Nonetheless, the data presented here show that it is possible to express a functional mAb as an IgA in NHPs, and this will inform future studies, which will need to achieve higher IgA expression levels and sample tissues at earlier timepoints post-transfusion to address these questions.

In contrast to the IgA2 data, mRNA-derived ePGDM1400v9 IgG1 expressed quite well, with a half-life comparable to the one observed for recombinant ePGDM1400v9 in NHPs (9.07 +/- 4.11 d, IAVI, unpublished data) and the parent mAb PGDM1400 in humans (28). Comparison of the serum concentration and neutralization titers observed here to those reported previously with the recombinant PGDM1400 parent antibody in IgG1 format in murine challenge and human clinical studies suggests that the mRNA-derived mAb reached expression levels that should be protective (28, 29). Indeed, considering a 90% Prevention Efficacy (PE) could be reached with a Predictive Titer PT_80_ of 200 (30) and knowing ePGDM1400v9 IgG1 has a mean IC80 of 0.08 µg/mL against contemporaneous clade C viruses from the AMP trials (IAVI, unpublished data), ePGDM1400v9 IgG serum protective titer would be 16 ug/mL against such panel of probable exposing viruses, which in this study correspond to >42 and >70 days for both IgG1 recipients (**Figure 1B**). We also observed detectable ePGDM1400v9 IgG1 in all tissues (**Figure 1D**) however, tissues were not perfused before extraction, so the presence of any antibody represents both the vasculature and the tissue.

Altogether, these results demonstrate that mRNA-LNPs can generate functional ePGDM1400v9 IgG1 *in situ* at levels expected to be protective. This could have important implications for the prophylactic and therapeutic use of HIV bnAbs. HIV bnAbs are actively being explored as prophylactics against HIV infection, particularly in the context of mother-to-child transmission at birth. Though recent clinical studies suggest that an antibody cocktail with sufficient viral coverage could prevent HIV infection (29–32), clinical implementation of such a therapeutic cocktail is currently cost-prohibitive. Since mRNA-LNPs bypass the expensive production of recombinant protein, this work provides an important proof-of-concept for mRNA delivery of HIV bnAbs in an NHP model. Future work can explore the delivery antibody cocktails by mRNA LNPs in NHPs and the protective efficacy of those cocktails against HIV/SIV infection in adult and infant macaques.

## Methods

### Recombinant antibody proteins

Recombinant ePGDM1400v9 IgA2 was purchased from GeneArt (ThermoFisher) whereby IgA2 protein was purified from transient transfection of a 4:4:1 ratio of HC to LC to JC by weight in Expi293 with CaptureSelect IgA Affinity matrix.

Recombinant ePGDM1400v9 IgG1 was produced via transient transfection of Expi293 cells using standard methods. To summarize, plasmids encoding for codon-optimized constructs of ePGDM1400v9 heavy and light chains were transfected at a 1:1 ratio by weight. Five days post-transfection, the antibody was purified by protein A/G affinity resin (Cytiva), and monodispersity was confirmed via size exclusion chromatography (SEC) using a Superdex S200 column (Cytiva). The ePGDM1400v9 Fab (with c-terminal His and FLAG tags) was performed using the same procedure, except that purification was performed using a Ni affinity column.

The anti-idiotype antibody aPGDM01 was raised against ePGDM1400v9 Fab using a commercial service. This antibody was validated by SPR, ELISA, and ECLIA (**Supplementary Figures S1A, E, F**). It was produced as either a Fab or a Murine IgG2a via transient transfection into Expi293 cells as described above for ePGDM1400v9 IgG1.

### mRNA transfection

EXPI293 cells were transiently transfected with the corresponding mRNA encoding for ePGDM1400v9 IgG1 or IgA2 using *trans* IT-mRNA Transfection Kit (Mirus Bio LLC) per the manufacturer’s recommendations. For IgG, this required co-transfection of HC and LC at a 2:1 ratio by weight, whereas for IgA, the transfection consisted of HC, LC, and JC transfections at a 4:4:1 ratio by weight. EXPI293 cells were diluted to 1x10^6^ cells/mL with EXPI293 Expression Medium (ThermoFisher). Using a ratio of 1 μg mRNA per 1 mL of culture, mRNA was first added to Opti-MEM I SFM (Gibco), followed by addition of TransIT-mRNA transfection reagent (Mirus Bio) at a 1:2 ratio of mRNA to TransIT. The complex was allowed to form for 3 min before addition to the suspension culture. The quantity of antibody in the resultant supernatants was measured by ELISA.

### Generation of modified mRNA and LNPs

Sequence-optimized mRNA encoding functional IgA monoclonal antibodies were synthesized *in vitro* using an optimized T7 RNA polymerase-mediated transcription reaction with complete replacement of uridine by N1-methyl-pseudouridine (33). The reactions included a DNA template containing an open reading frame flanked by 5′ untranslated region (UTR) and 3′ UTR sequences and was terminated by an encoded polyA tail. Ligation reactions were performed using T4 RNA Ligase I (New England Biolabs) according to the manufacturers recommended conditions. mRNA was purified by dT affinity chromatography, buffer exchanged by ultrafiltration into 2 mM sodium citrate, sterile filtered, assayed for purity, and stored frozen at –20°C for further use.

Lipid nanoparticle-formulated mRNA was produced through a modified ethanol-drop nanoprecipitation process as described previously (34). Briefly, ionizable, structural, helper, and polyethylene glycol lipids were mixed with mRNA (2:1 HC to LC by weight for IgG; 4:4:1 HC to LC to JC by weight for IgA) in acetate buffer, pH 5.0, at a ratio of 3:1 (lipids:mRNA), as performed in (5). The mixture was neutralized with Tris-Cl, pH 7.5, sucrose was added as a cryoprotectant, and the final solution was sterile filtered. Vials were filled with formulated LNP and stored frozen at –70°C until further use. The drug product underwent analytical characterization, which included the determination of particle size and polydispersity, encapsulation, mRNA purity, double stranded RNA content, osmolality, pH, endotoxin and bioburden, and the material was deemed acceptable for *in vivo* study.

### Research animals and study design

Eight (6 male and 2 female) adult Indian-origin rhesus macaques were infused with liposomes containing mRNA expressing ePGDM1400v9-LS. Four macaques were infused with the mRNA encoding the antibody in an IgG1 format and four macaques were infused with the mRNA encoding the antibody in an IgA format. Blood and mucosal secretions were collected at multiple time points to collect pharmacokinetic data for each antibody. Rhesus macaques were housed at the Wisconsin National Primate Research Center (WNPRC). All procedures were approved by the University of Wisconsin-Madison College of Letters and Sciences and the Office of the Vice Chancellor for Research Institutional Animal Care and Use Committee (IACUC protocol number G006597). The animal facilities of the WNPRC are licensed by the US Department of Agriculture and accredited by AAALAC. Animals were monitored twice daily by veterinarians for any signs of disease, injury, pain, distress, or psychological abnormalities and treated as recommended by the veterinarian. Animals were euthanized at either 5- or 70-days post mRNA infusion. Animals were euthanized as recommended by the Panel on Euthanasia of the American Veterinary Medical Association by first administering general anesthesia (ketamine ≥15 mg/kg intramuscularly (IM)), then administering an intravenous (IV) overdose of sodium pentobarbital (≥ 50 mg/kg). After euthanasia tissues were taken at necropsy and either flash frozen, frozen into OCT blocks or embedded into FFPE blocks.

### LNP-mRNA infusion

LNP-mRNA infusion was performed under anesthesia (ketamine plus dexmedetomidine). Animals were prophylactically dosed intramuscularly with 1 mg/kg diphenhydramine 30 minutes prior to the infusion to prevent anaphylaxis. The animals were fitted with a catheter and the infusion of the mRNA was performed intravenously at a dose of 1 mg/kg over approximately a 1 h duration. Animals were closely monitored for reactions following the infusion and during anesthesia recovery. Blood was drawn longitudinally following infusion to monitor antibody levels.

### Sample collection, processing, and cryopreservation

Blood was collected from animals prior to the mRNA infusion, before and immediately after infusion, and then every 2 days through day 13 and then weekly for 5 weeks. Blood was collected into EDTA tubes and SST tubes and centrifuged twice to remove all cells and collect plasma and serum, respectively. Plasma and serum were stored at -80°C.

Rectal and nasal secretions were sampled onto pre-weighed Weck-Cel sponges (Fisher Scientific) while the animals were under anesthesia (ketamine up to 7 mg/kg IM and up to 0.03 mg/kg dexmedetomidine IM, reversed with 0.3 mg/kg atipamezole (IV or IM)). Sponges were pre-moistened with sterile PBS and placed atraumatically into the rectal or nasal cavity for five minutes. Sponges were removed and placed into a sterile microcentrifuge tube. The post-collection swab was weighed and then the stick cut off, so the sponge fit inside the microcentrifuge tubes. Sponges were stored at -80°C until tested.

### Tissue processing

NHP tissues were collected at necropsy and shipped to Charles River Laboratories for processing. Tissues pieces were weighed and cut to be approximately between 5 and 20 mg. Samples were added to pre-chilled Matrix D tubes (MP Biomedicals #6913050) and 200 uL of Tris lysing buffer (MSD #R60RTX) was added prior to placing the tubes on the FastPrep-24 homogenizer (MP Biomedical #116004500). For all tissues, the FastPrep-24 homogenizer was set to a speed of 6 meters/second for a time of 30 seconds. Following the first run, tubes were placed on wet ice for at least 5 minutes after which the FastPrep-24 homogenization was repeated 2 more times, with 5-minute incubation on ice in between. All samples were visually inspected for complete homogenization and then spun in a centrifuge at 20000 x g at 5°C for 20 minutes. Supernatants were transferred to new tubes on wet ice and the spin was repeated. Following the final spin, supernatants were transferred to new tubes and stored at –80°C until use. Collected tissues used for BioD analysis included Lung, vaginal, Rectal, Ascending Colon, Duodedum, Jejunum, Inguinal and teal LN, Spleen, Liver.

### Ig extraction from NHP nasal and rectal wecks

The neutralization assay was performed on antibody extracted from nasal and rectal wecks. Briefly, each weck tubes received 150 µL of weck buffer (1xPBS, 10% normal goat serum and, for rectal wecks only, protease inhibitor) in which they incubated for 2 hours at RT. Each weck is then transferred to a new tube using forceps, and centrifugated at max speed for 5 min before being squeezed with tweezers, to extract as much remaining buffer as possible.

The recovered volume will be combined with the first extraction volume.

### Serum and tissue Ig concentration measurement

Serum and tissue monoclonal IgG1 and IgA2 were measured using MSD ECL. The assay (**Supplementary Figures S1E, F**) was standardized using ePGDM1499v9-free NHP serum spiked with recombinant ePGDM1400v9 (either human IgG1 or human IgA2). 96 well MSD QuickPlex Standard plates (MSD Cat# L55XA) were coated in PBS with anti-human IgG CH2 domain (BioRad MCA5748G) for IgG and goat anti human kappa (Southern Biotech 2042-01) for IgA2. All coated plates were stored at 2-8°C until use. Prior to use, coated plates were removed from 2-8°C and allowed to warm to room temperature. Assay plates were washed 3 times with 300 µL per well of ELISA wash buffer (0.05% Tween 20 in TBS-T). Plates were blocked with 150 µL per well of 5% MSD Blocker A (MSD R93AA-1), sealed and incubated on a plate shaker (BoekelScientific 130000–2 microplate shaker) at room temperature for 2 h. Fresh standards and quality controls were thawed on the bench, briefly spun down and vortexed. ePGDM1400 IgG1 and IgA2 recombinant proteins served as standards for the respective assay. Standards for all serum ECL assays were diluted in heat inactivated normal rhesus macaque serum and for tissue ECL assays, standards were diluted in 5% Tris lysis buffer. For serum IgG1, standards ranged from 500 ng/mL to 1.95 ng/mL and for serum and tissue IgA2, standards ranged from 500 ng/mL to 0.98 ng/mL. Tissue IgG1 standards started at 300 ng/mL and were diluted down to 0.0457 ng/mL. All standards and samples were diluted 1:10 (for serum ECL) and 1:20 (for tissue ECL) in low cross buffer (Boca Scientific 100-500) for a final volume of 100 μL. Following blocking incubation, plates were washed 3x with ELISA washer buffer and 30 µL of diluted standards and samples were added to the plate in duplicate. Plates were sealed and incubated at RT on a plate shaker for 1 h. After primary incubation, plates were washed 3 times with ELISA wash buffer and 30 µL per well of secondary reagent was added. For both IgG1 and IgA2, a sulfotagged aPGDM01 diluted in 1% MSD Blocker A buffer was used (1:4000 dilution IgG1, 1:500 dilution IgA2). Plates were incubated at room temperature on a plate shaker for 1 hour. Following incubation, plates were washed 3 times with ELISA wash buffer and 150 µL of MSD Gold Read Buffer A was added to each well (MSD R92TG). All plates were read within 10 min. Sample concentrations were extrapolated from the standard curve using Watson 7.6.1 analysis software and a 5PL regression curve.

### Antibody half-life calculations

Antibody half-life was calculated for NHPs not necropsied at 4 d post infusion based on the measured serum concentration of ePGDM1400v9 using exponential decay functions provided by GraphPad Prism^®^. The plateau was held at the limit of detection for the ECL measurements. Values prior to 1 d post-infusion were excluded from analysis, as the concentration of ePGDM1400v9 was still increasing during this period. Values were also excluded in cases of expected anti-drug antibody responses (ADA), as this made calculation of an accurate half-life impossible. ADA was presumed in cases where there was a >50-fold decrease in serum concentration between two time-points that brought the concentration down to the limit of detection, and this was applied for RM8 at 11 d post-infusion and RM7–56 d post-infusion. For all NHPs, a biphasic exponential decay function was used to calculate half-life, as mAbs typically exhibit biphasic decay kinetics (19). A biphasic exponential decay function was found to fit the observed data well for all animals (**Supplementary Figure S3**, **Supplementary Table S2**), and this was used to calculate the half-life of mRNA-derived ePGDM1400v9 in NHPs receiving the IgA2 isotype.

### Nasal and rectal weck Ig concentration measurement

Nasal and rectal wecks were stored frozen at –80 °C until processed. All wecks were inspected prior to processing for presence of feces and/or blood and this data was recorded. For both rectal and nasal wecks, there were two wecks per monkey per time point. Thawed rectal wecks were soaked in 150 μL of PBS + 10% normal goat serum (ThermoFisher) and 1 protease inhibitor (Thermo Scientific) per 50mL of buffer for 2 h at room temperature. Thawed nasal wecks were soaked in 150 μL of PBS + 10% normal goat serum for 2 h at room temperature. Following incubation, wecks were taken out of buffer and placed with weck facing up in a new 1.5 mL eppendorf tube and spun at 14,000 rpm for 5 min to capture to dry out the weck and collect the fluid at the bottom of the tube. After centrifuging, tweezers were used to squeeze any remaining liquid out of the wecks and dispose of the weck. Centrifuge tubes again to collect all fluid at the bottom of the tube. Weck fluid was combined for the two wecks for each monkey to assess antibody levels in ELISA.

To assess levels of antibody in processes wecks, half-area 96 well plates (Corning Costar) were coated with 25 µL per well of 4 µg/mL of the anti-idiotype mAb aPGMD01 (as a murine IgG2a) overnight at 4 °C. Plates were washed using an automated plate washer (Biotek) with 0.05% PBS-Tween (PBS-T) and were subsequently blocked for 2 h with 50 µL per well of Superblock PBST (ThermoFisher). Following blocking, plates were washed again using an automated plate washer and incubated with weck samples at 4°C overnight. Weck samples were added straight onto the plate and were not diluted. Purified recombinant ePGDM1400v9 IgG1 or IgA2 was used as standards on all plates. The next day, plates were washed with an automated plate washer (Biotek) and incubated with 25 µL per well either goat anti human IgA (alpha chain, cross adsorbed) HRP (Life Technologies, 1:10,000) or goat anti human H+L chain NHP cross-adsorbed HRP (American Qualex, 1:10,000) for 1 h at room temperature. Plates were subsequently washed and incubated with 25 µL per well of SureBlue TMB 1-C substrate (Fisher Scientific) for 5 min. The reaction was stopped with 25 µL per well of TMB Stop solution (SeraCare) and read at an absorbance of 450 nM on a SpectraMax ABS Microplate reader. Absolute quantities of human antibody in wecks were extrapolated in GraphPad Prism 9 using a standard curve that was generated with the appropriate purified isotype version of ePGDM1400v9.

### Neutralization Ig assay

Rhesus macaque sera or extracted Ig and control Igs were serially diluted in 96-well plates (Corning) and DMEM (Gibco) completed with 10% FBS (Gibco), 0.1 mg/mL Penicillin-Streptomycin (Gibco) and 2 mM L-Glutamine 2 mM (Gibco). 25 µL of each serum or antibody dilution were incubated with 25 µL of pseudotype virus supernatant for 1 h at 37 °C, prior addition of 50 µL per well TZMbl cells at 0.2 million cells/mL in suspension in DMEM complete (assay plate: Corning). The serum or antibody, virus and cells mix were incubated for 72 h at 37°C prior medium aspiration, cell lysis and luciferase substrate addition (Luciferase Assay kit, Promega). Light intensity – reporter for infection - is measured with a luminometer (Biotek Synergy). The percentages of neutralization were calculated with excel and ID80 (dilution required to reach 80% neutralization against a given virus) were defined with GraphPad Prism.

### Surface plasmon resonance

SPR measurements were performed on a Biacore 8K instrument at 25 °C. All experiments used HBS-EP+ (0.1M HEPES pH 7.6, 150 mM NaCl, 3 mM EDTA, 0.0005% (v/v) Surfactant P20) as a mobile phase at a flow rate of 30 µL/min. Anti-mouse IgG (Fc-specific) antibody (Cytiva Cat. No. BR100838) was immobilized on a Series S CM-3 sensor chip (Cytiva) using standard NHS/Edc coupling in accordance with the manufacturer’s instructions. A reference surface was generated using the same methods.

To validate that aPGMD01 bound ePGDM1400v9 in Fab, IgG1, and IgA2 formats, aPGMD01 was produced as mouse IgG2a mAbs and captured at 10 nM onto the anti-mouse IgG coated chips described above. A recombinant mouse IgG2a mAb that did not have detectable binding to ePGDM1400v9 by ELISA was used as a negative control. A concentration series of analyte (either recombinant ePGDM1400v9 Fab, human IgG1, or human IgA2) was injected across the antibody and control surfaces for 2 min followed by a 10 min dissociation step using a single-cycle method. Regeneration of the surface between injections of analyte was accomplished using 4-min injections of 10 mM Glycine (pH 1.7). Data analysis was performed using BIAEvaluation software (Cytiva) and Prism (Graphpad).

### Purification of mRNA-produced ePGDM1400v9 from NHP sera/plasma

To purify mRNA-derived ePGDM1400v9 antibodies from NHP sera/plasma for glycan analysis, recombinant aPGDM01 was produced as a mouse IgG2a and coupled to CNBr resin (Cytivia Cat. No. 17043001) using the manufacturer’s protocol, except that PBS (pH 7.4) was used as coupling buffer.

Sera and/or plasma that had not been used for other experiments was pooled prior to applying it to the aPGDM01-coupled column. For purifying ePGDM1400v9 IgG, all available serum samples up to 42 d post-transfusion were pooled. For purifying ePGDM1400v9 IgA, plasma and sera from all animals up to 14 d for RMA3, up to 11 d for RMA4, and up to 5 d for all four NHPs based on sample availability and measured IgA from the PK measurements.

For the purifications, 250 µL of aPGDM01-coupled resin was washed with 10 mL of 1X PBS pH 7.4. Then, the pooled serum/plasma fractions were applied to the column by gravity flow. Next, the column was washed with 5 mL of salt wash buffer (0.1 M Tris pH 8.0, 500 mM NaCl), followed by a 5 mL detergent wash (1X PBS pH 7.4, 0.1% n-dodecyl-ß-D-maltoside (DDM)). A final wash of 5 mL 1X PBS pH 7.4 was then applied before eluting with 650 µL 0.1 M sodium citrate buffer, pH 3.0. The pH was neutralized by the addition of 350 µL 1 M Tris pH 8.0. The purified antibody was stored at 4 °C overnight. In the morning, the quality and quantity of the purified antibodies were confirmed by a Coomassie gel and a PGDM1400v9-specific ELISA (using plates coated with aPGDM01). The remaining preparations were then aliquoted and frozen at -20 °C until use.

### Glycosylation analysis of mRNA-produced ePGDM1400v9 purified from NHP plasma/sera

To analyze the site-specific glycosylation of mRNA-derived Ig, the pooled remaining protein was incubated for 1 h in 50 mM Tris/HCl, pH 8.0, containing 6 M of urea and 5 mM dithiothreitol (DTT). The sample was then incubated in the dark for 1 h with 20 mM iodoacetamide (IAA) to alkylate the protein. To remove the residual IAA, 20 mM DTT was added, and the sample was incubated for an additional 1 h period. Following buffer-exchange into 50 mM Tris/HCl, pH 8.0 using Vivaspin columns (10 kDa molecular weight cutoff). The proteins were then digested separately overnight at 37°C with Trypsin (Promega) in a 1:30 (w/w) ratio.

Following digestion, a heated vacuum centrifuge set at 30°C was used to dry down the peptides, which were then resuspended in 250 µL 0.1% TFA. Desalting and peptide enrichment was performed using an Oasis HLB desalting 96-well µElution plate (Waters) attached to a vacuum manifold. One well per digest was first equilibrated with 200 µL acetonitrile (ACN), followed by 200 µL 0.1% TFA. The peptides were loaded onto the elution plate at 1 mL/min, and the wells were then washed, first with 800 µL 0.1% TFA, followed by 200 µL of LC-MS grade H_2_O. The peptides were eluted by the addition of 70% ACN in 0.1% TFA and dried down again before being re-suspended in 0.1% formic acid. The peptides were combined and analyzed by nanoLC-ESI MS with a Vanquish Neo HPLC (Thermo Fisher Scientific) system coupled to an Orbitrap Eclipse mass spectrometer (Thermo Fisher Scientific). A μPAC™ Neo HPLC ColumnC18 column (75 µm x 110 cm) was used to separate the peptides. A trapping column (PepMap 100 C18 3 µM 75 µM x 2 cm) was used in line with the LC prior to separation with the analytical column. For LC separation, buffer A consisted of 0.1% formic acid and 80% acetonitrile in 0.1% formic acid. The LC conditions were as follows: 280-minute linear gradient consisting of 5-40% B in 0.1% formic acid over 240 minutes. The %B was then increased to 95% over 15 minutes and held for another 15 minutes before reducing the %B to 5%. The flow rate was set to 300 nL/min. The spray voltage was set to 2.5 kV. The ion transfer tube temperature was set to 275°C. The scan range was 300−2000 m/z. Stepped HCD collision energy was set to 15, 25 and 45% and the MS2 for each energy was combined. Precursor and fragment detection were performed using an Orbitrap at a resolution MS1 = 120,000. MS2 = 30,000. The AGC target for MS1 was set to standard and injection time set to auto which involves the system setting the two parameters to maximize sensitivity while maintaining cycle time. Glycopeptide fragmentation data were extracted from the raw MS files using Byos (Version 4; Protein Metrics Inc). The glycopeptide fragmentation data were evaluated manually for each glycopeptide. The peptide was scored as true-positive when both the oxonium ions corresponding to the identified glycan and the correct b and γ fragment ions were observed. The Protein Metrics 305 N-glycan library with sulphated glycans added manually, was used to search the MS data. The relative amounts of each glycan at each site in addition to the unoccupied proportion was determined by comparing the extracted chromatographic areas for different glycoforms with an identical peptide sequence. A 1% False discovery rate (FDR) was applied, and the precursor mass tolerance was set at 4 ppm, and 10 ppm for fragments. All charge sates for a single glycopeptide were summed. Glycans were categorized according to the composition detected. The assigned compositions can be found in **Supplementary File** - Glycopeptide analysis.

## Data Availability

The original contributions presented in the study are included in the article/**Supplementary Material**. Further inquiries can be directed to the corresponding authors.

## Supplementary material

Supplementary Figure 1 Anti-idiotype anti-ePGDM1400v9 mAb aPGDM01 enables ePGDM1400v9 IgG1 and IgA2 quantitation extrapolation via ECL. **(A)** Single-cycle SPR data using chips coated with aPGMD01 showing that the anti-idiotype mAb aPGMD01 binds ePGDM1400v9 as a Fab, IgG1, and IgA2. A negative control protein was run for each measurement and did not exhibit binding over background. **(B)** SEC trace of recombinant ePGDM1400v9 IgA2, showing distinct monomer and dimer peaks. Fractions were collected from indicated peaks, and dimer or monomer assignment was made based on the mass of each species on a Coomassie gel. Data acquired on a Superose 6 column in PBS (pH 7.4). **(C)** Acrylamid gel of ePGDM1400v9 IgG, IgA2 recombinant, IgA2 monomer and dimer discriminated by SEC **(D)** ELISA data showing binding of ePGDM1400v9 to plates coated with aPGDM01 mAb. Recombinant ePGDM1400v9 was diluted in PBS. Note that signal for IgG1 and IgA2 are not directly comparable, as different secondary antibodies were used. **(E)** Quantification of recombinant eGPDM1400v9 IgG1 diluted in non-mRNA infused NHP serum using the final aPGMD01-based ECL assay used to measure mRNA-derived ePGDM1400v9 IgG1 from NHPs infused with LNPs. **(F)** Quantification of recombinant eGPDM1400v9 IgA2 diluted in non-mRNA infused NHP serum using the final aPGMD01-based ECL assay used to measure mRNA-derived ePGDM1400v9 IgA2 from NHPs infused with LNPs.

Supplementary Figure 2 Experimental design for assessment of mRNA-delivered ePGDM1400v9 in IgG1 or IgA2 isotype biodistribution *in vivo*. A total of 8 rhesus macaques received ePGDM1400v9-IgG1 or ePGDM1400v9-IgA2 encoding mRNA at 1 mg/kg i.v. at day 0. Half (n=2) animals per groups were necropsied at day 4 and the second half at day 70. Plasma, Serum, nasal and rectal wecks were collected at -6 h, 0 h, and +6 h, and 1 d, 2 d, 4 d, and for the 4 remaining animals, also at 7 d, 9 d, 11 d, 14 d, 22 d, 28 d, 42 d, 56 d and 70 d.

Supplementary Figure 3 PK fits for mRNA-delivered ePGDM1400v9 as IgG1 or IgA2 isotypes in animals not necropsied at 4 d post-infusion. For NHPs not necropsied at 4 d post-infusion, mAb half-life was calculated for both the IgG1 isotype **(A)** and the IgA2 isotype **(B)**. To calculate half-life, measurements of ePGDM1400v9 in serum was fit with either with a monophasic (colored dotted line) or a biphasic (solid colored line) decay curve. Points at or below the ECL limit of detection (dotted black line) were excluded from analysis (see methods).

Supplementary Table 1 mRNA-delivered ePGDM1400v9 in IgG1 or IgA2 isotype quantification in serum, nasal and rectal wecks and different tissues.

Supplementary Table 2 Serum half-life calculations for animals not necropsied at day 4. Best fit values for serum half-lives of ePGDM1400v9 as an IgG1 or IgA2 based on ECL measurements of serum antibody concentration.

Supplementary Table 3 Longitudinal neutralization ID_50_ of ePGDM1400v9 in IgG1 or IgA2 isotype from the NHP serum samples.

Supplementary Table 4 Extrapolated IC_50_ (in µg/mL) at day 1 and day 4 post-mRNA transfusion, calculated from serum concentrations and neutralization ID_50_, against T250–4 and CE1176 Env.

Supplementary Table 5 Site-specific glycosylation of IgG and IgA.
