# Supplementary figures and images for: Harnessing mRNA for the expression of monoclonal IgG and IgA in non-human primates
Source: Front Immunol. 2026 Jan 16;16:1700041. doi: 10.3389/fimmu.2025.1700041 (PMC12855044; doi:10.3389/fimmu.2025.1700041)

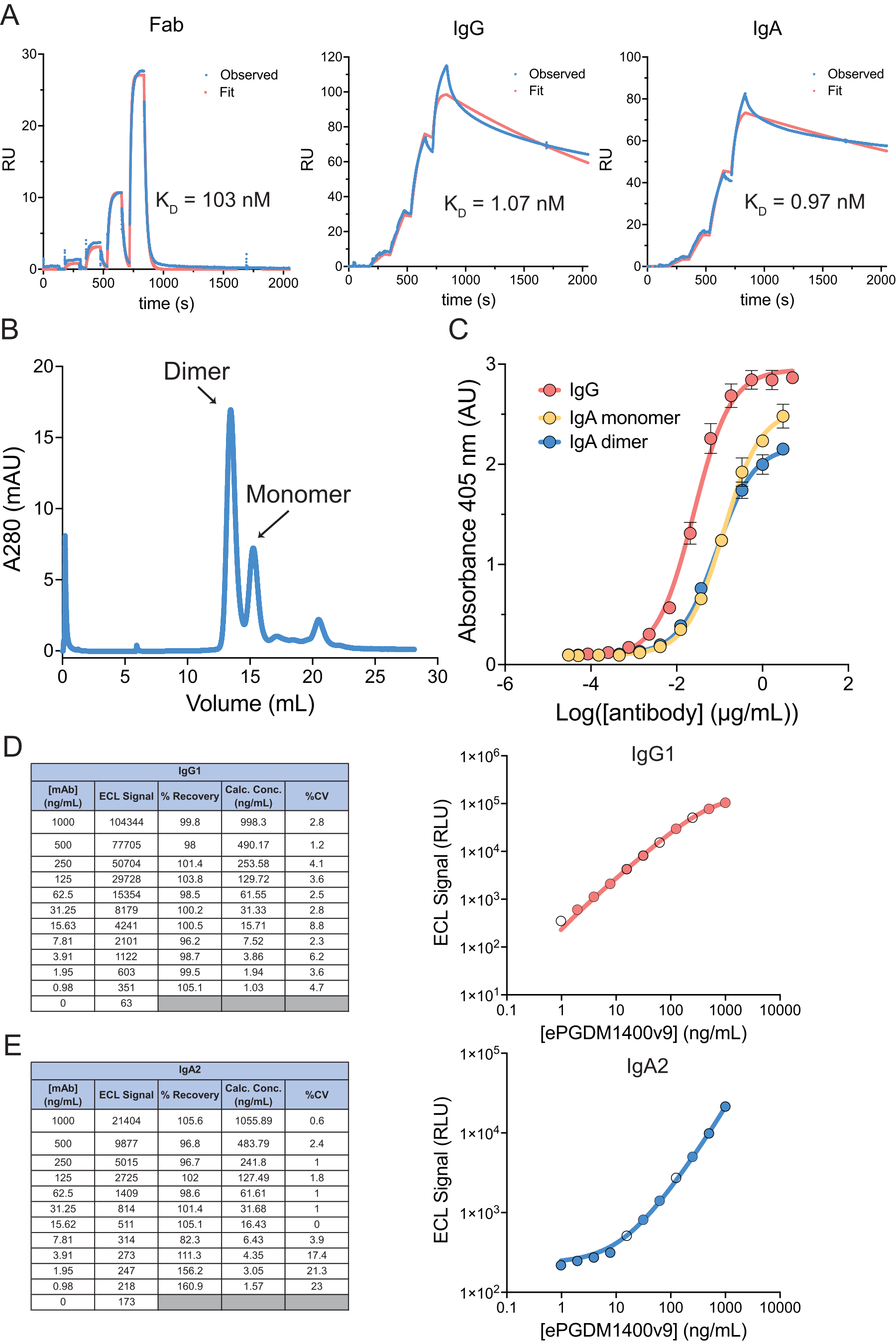

Supplement: Supplementary Figure 1 — Anti-idiotype anti-ePGDM1400v9 mAb aPGDM01 enables ePGDM1400v9 IgG1 and IgA2 quantitation extrapolation via ECL. (A) Single-cycle SPR data using chips coated with aPGMD01 showing that the anti-idiotype mAb aPGMD01 binds ePGDM1400v9 as a Fab, IgG1, and IgA2. A negative control protein was run for each measurement and did not exhibit binding over background. (B) SEC trace of recombinant ePGDM1400v9 IgA2, showing distinct monomer and dimer peaks. Fractions were collected from indicated peaks, and dimer or monomer assignment was made based on the mass of each species on a Coomassie gel. Data acquired on a Superose 6 column in PBS (pH 7.4). (C) Acrylamid gel of ePGDM1400v9 IgG, IgA2 recombinant, IgA2 monomer and dimer discriminated by SEC (D) ELISA data showing binding of ePGDM1400v9 to plates coated with aPGDM01 mAb. Recombinant ePGDM1400v9 was diluted in PBS. Note that signal for IgG1 and IgA2 are not directly comparable, as different secondary antibodies were used. (E) Quantification of recombinant eGPDM1400v9 IgG1 diluted in non-mRNA infused NHP serum using the final aPGMD01-based ECL assay used to measure mRNA-derived ePGDM1400v9 IgG1 from NHPs infused with LNPs. (F) Quantification of recombinant eGPDM1400v9 IgA2 diluted in non-mRNA infused NHP serum using the final aPGMD01-based ECL assay used to measure mRNA-derived ePGDM1400v9 IgA2 from NHPs infused with LNPs. [file Image1.tiff]

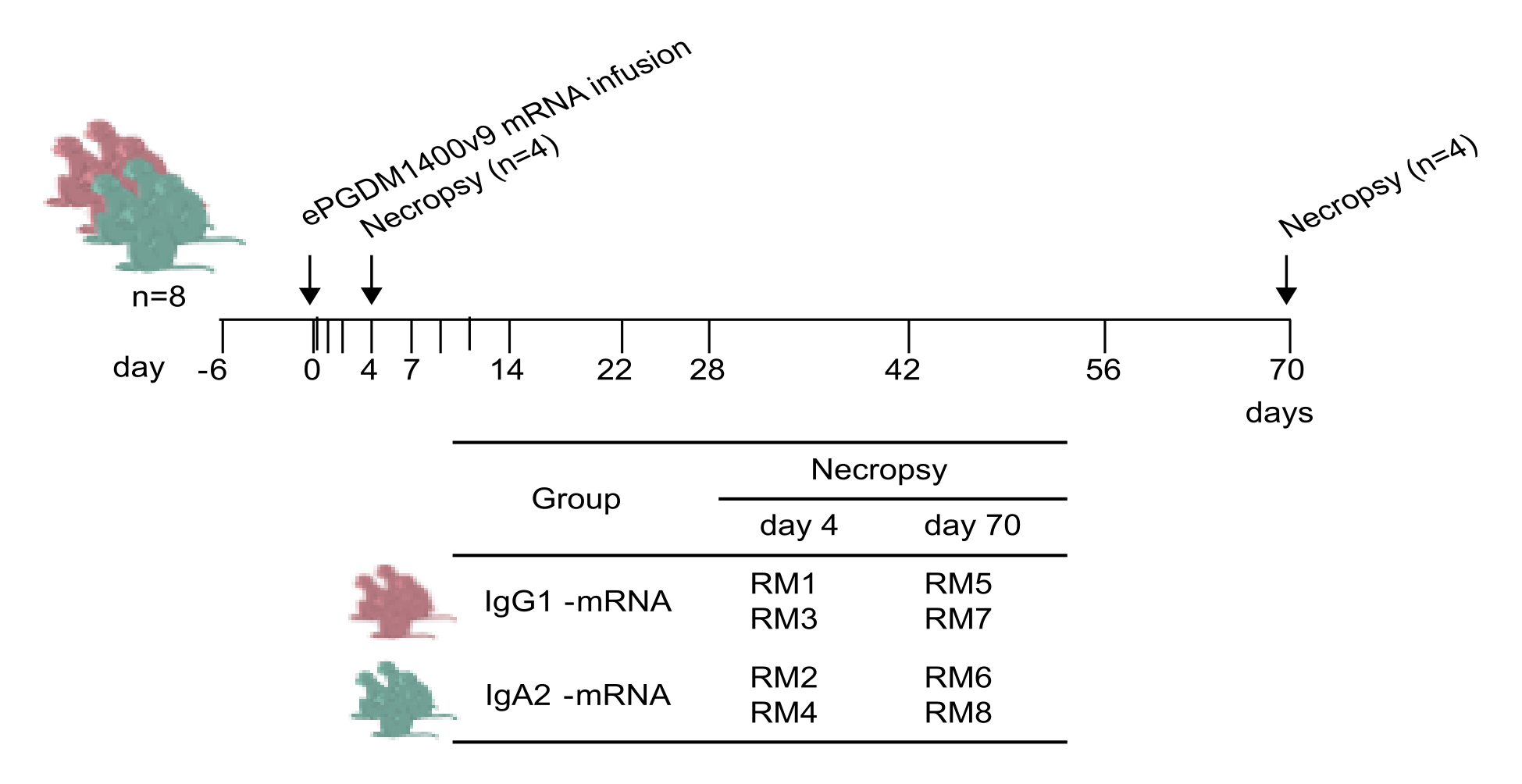

Supplement: Supplementary Figure 2 — Experimental design for assessment of mRNA-delivered ePGDM1400v9 in IgG1 or IgA2 isotype biodistribution in vivo. A total of 8 rhesus macaques received ePGDM1400v9-IgG1 or ePGDM1400v9-IgA2 encoding mRNA at 1 mg/kg i.v. at day 0. Half (n=2) animals per groups were necropsied at day 4 and the second half at day 70. Plasma, Serum, nasal and rectal wecks were collected at -6 h, 0 h, and +6 h, and 1 d, 2 d, 4 d, and for the 4 remaining animals, also at 7 d, 9 d, 11 d, 14 d, 22 d, 28 d, 42 d, 56 d and 70 d. [file Image2.tiff]

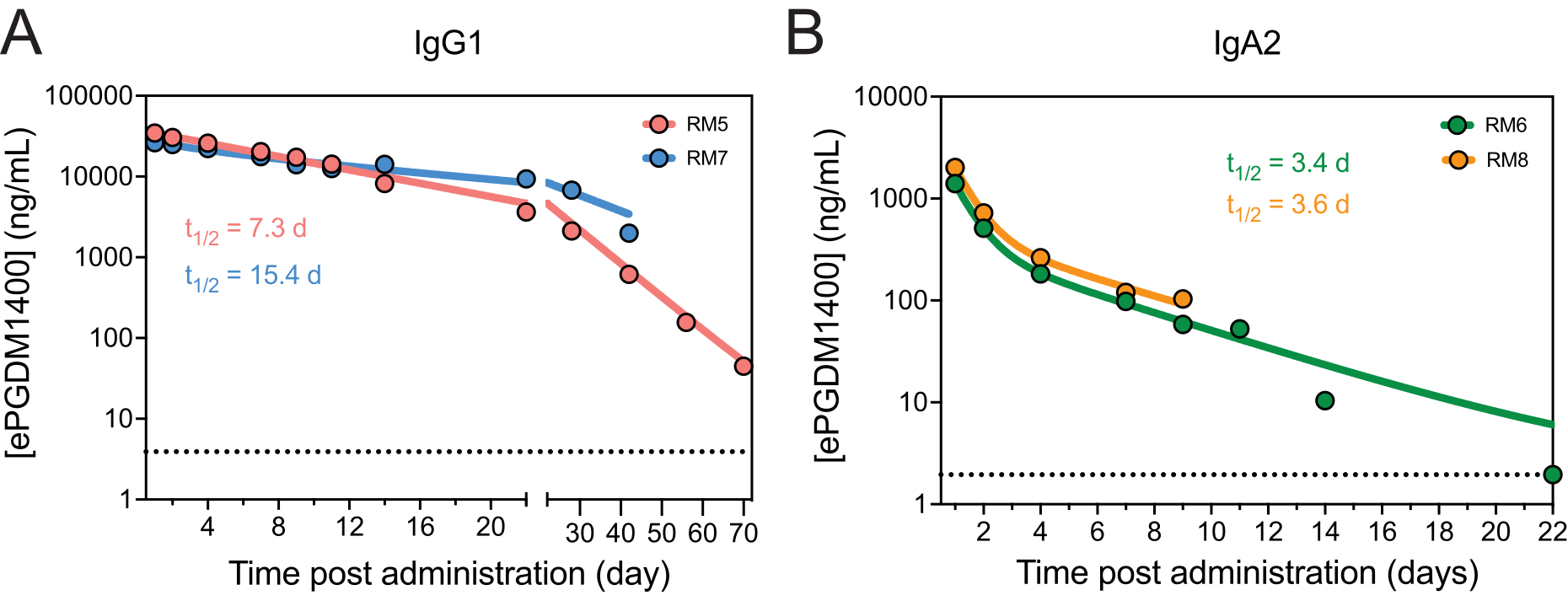

Supplement: Supplementary Figure 3 — PK fits for mRNA-delivered ePGDM1400v9 as IgG1 or IgA2 isotypes in animals not necropsied at 4 d post-infusion. For NHPs not necropsied at 4 d post-infusion, mAb half-life was calculated for both the IgG1 isotype (A) and the IgA2 isotype (B). To calculate half-life, measurements of ePGDM1400v9 in serum was fit with either with a monophasic (colored dotted line) or a biphasic (solid colored line) decay curve. Points at or below the ECL limit of detection (dotted black line) were excluded from analysis (see methods). [file Image3.tiff]

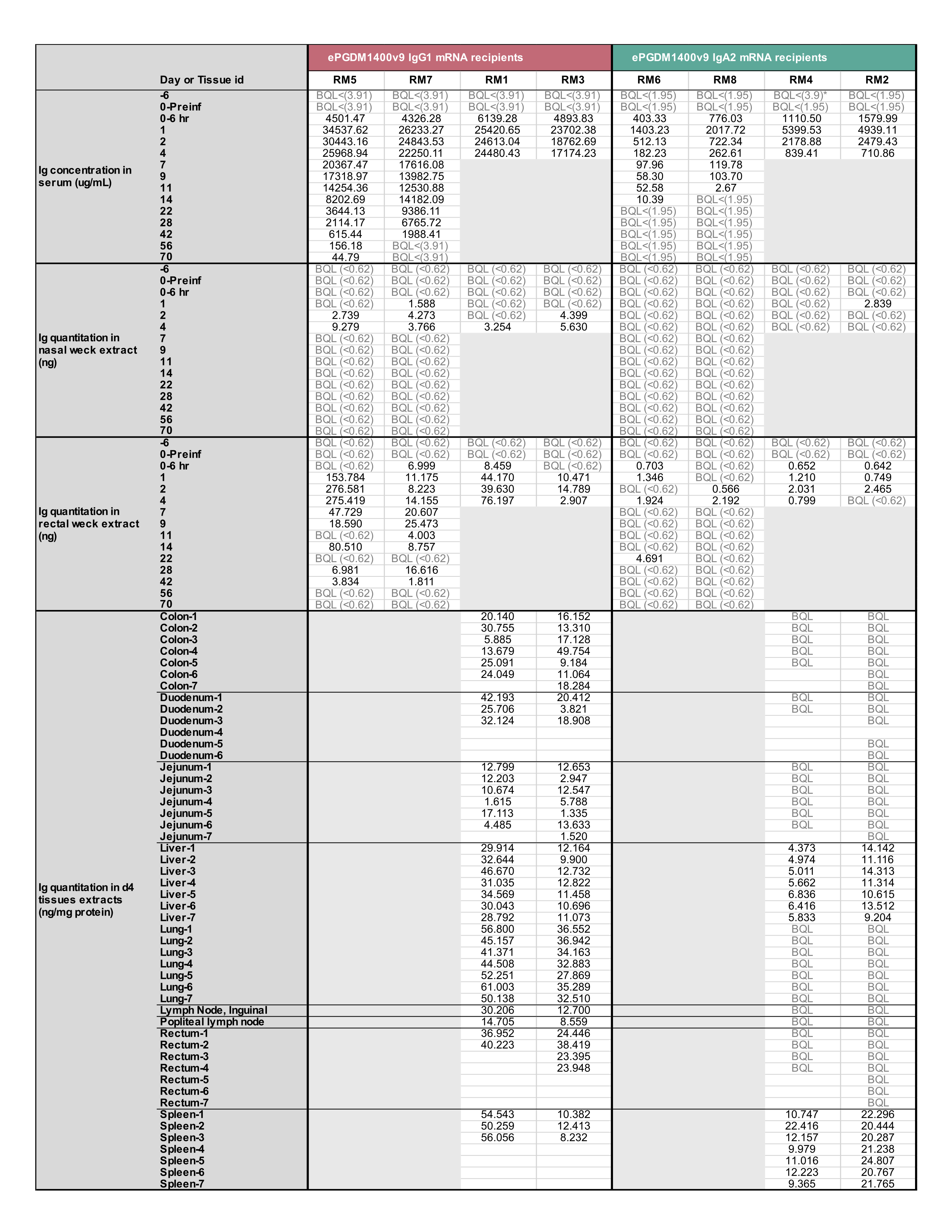

Supplement: Supplementary Table 1 — mRNA-delivered ePGDM1400v9 in IgG1 or IgA2 isotype quantification in serum, nasal and rectal wecks and different tissues. [file Image4.tiff]

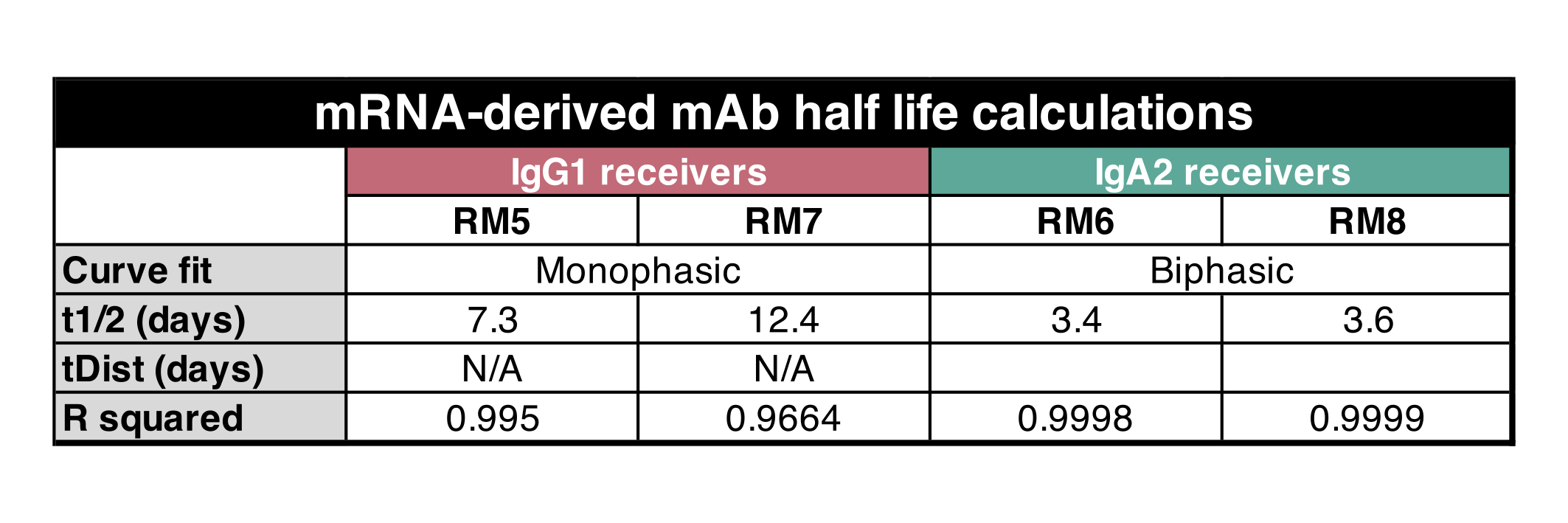

Supplement: Supplementary Table 2 — Serum half-life calculations for animals not necropsied at day 4. Best fit values for serum half-lives of ePGDM1400v9 as an IgG1 or IgA2 based on ECL measurements of serum antibody concentration. [file Image5.tiff]

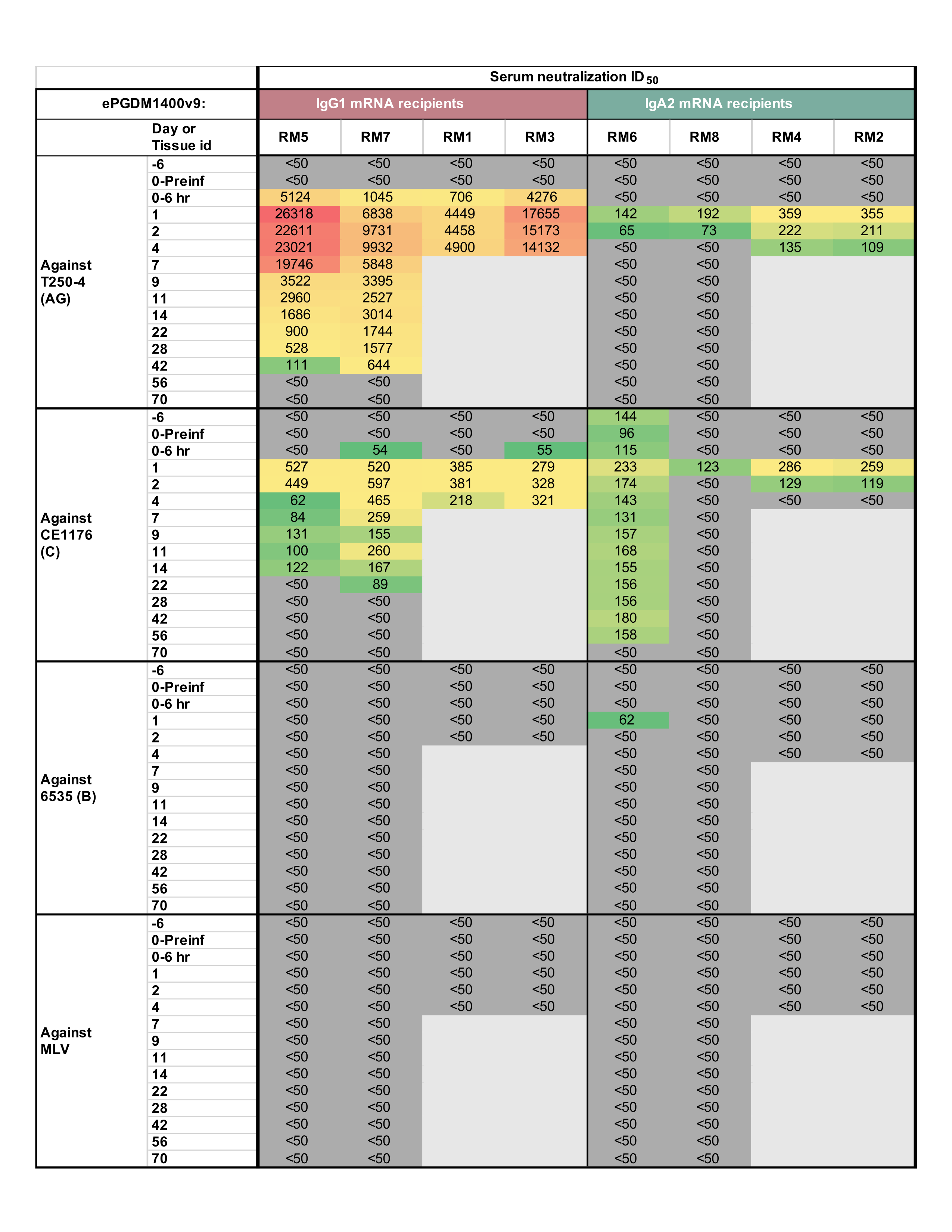

Supplement: Supplementary Table 3 — Longitudinal neutralization ID50 of ePGDM1400v9 in IgG1 or IgA2 isotype from the NHP serum samples. [file Image6.tiff]

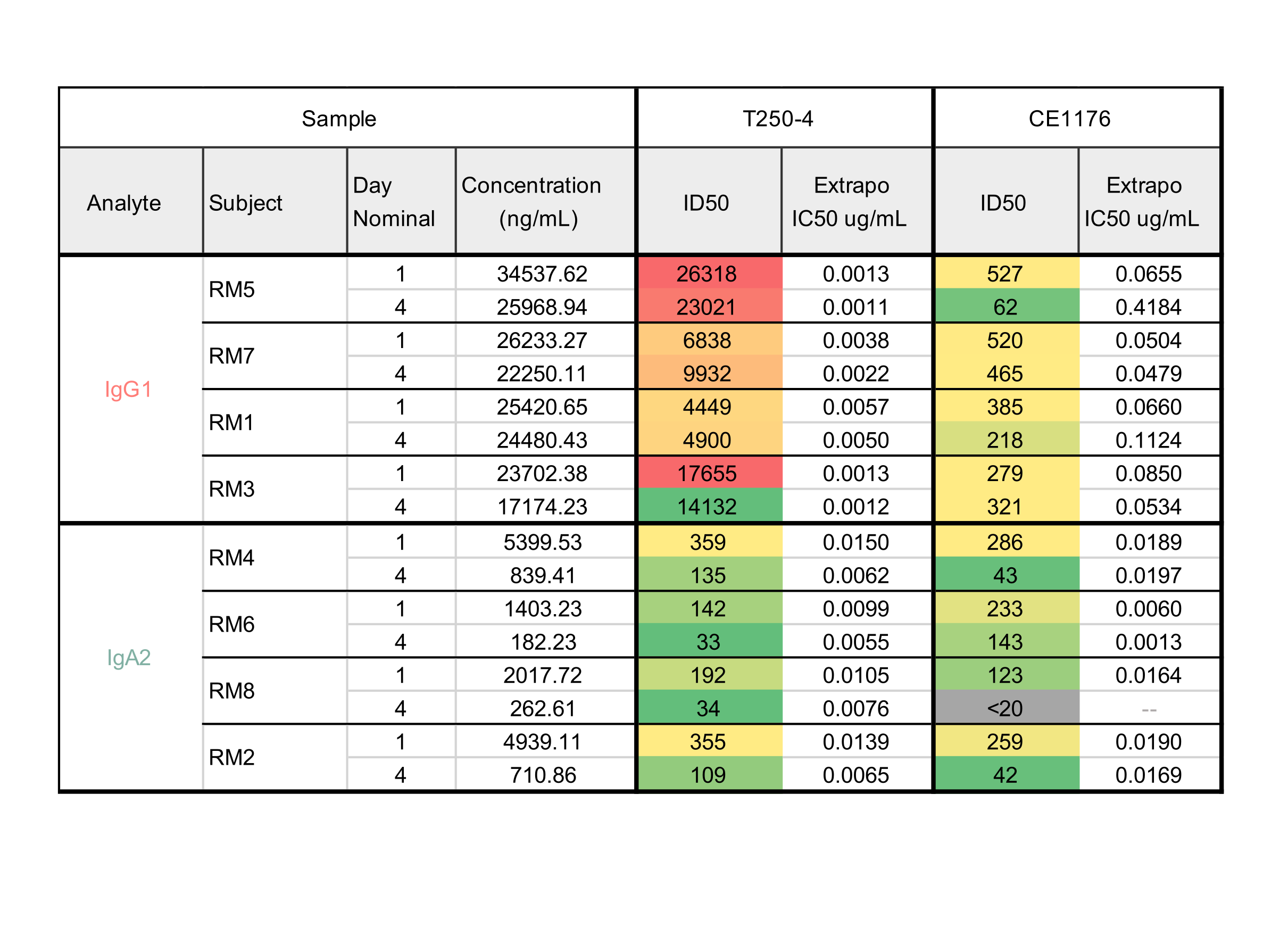

Supplement: Supplementary Table 4 — Extrapolated IC50 (in µg/mL) at day 1 and day 4 post-mRNA transfusion, calculated from serum concentrations and neutralization ID50, against T250–4 and CE1176 Env. [file Image7.tiff]

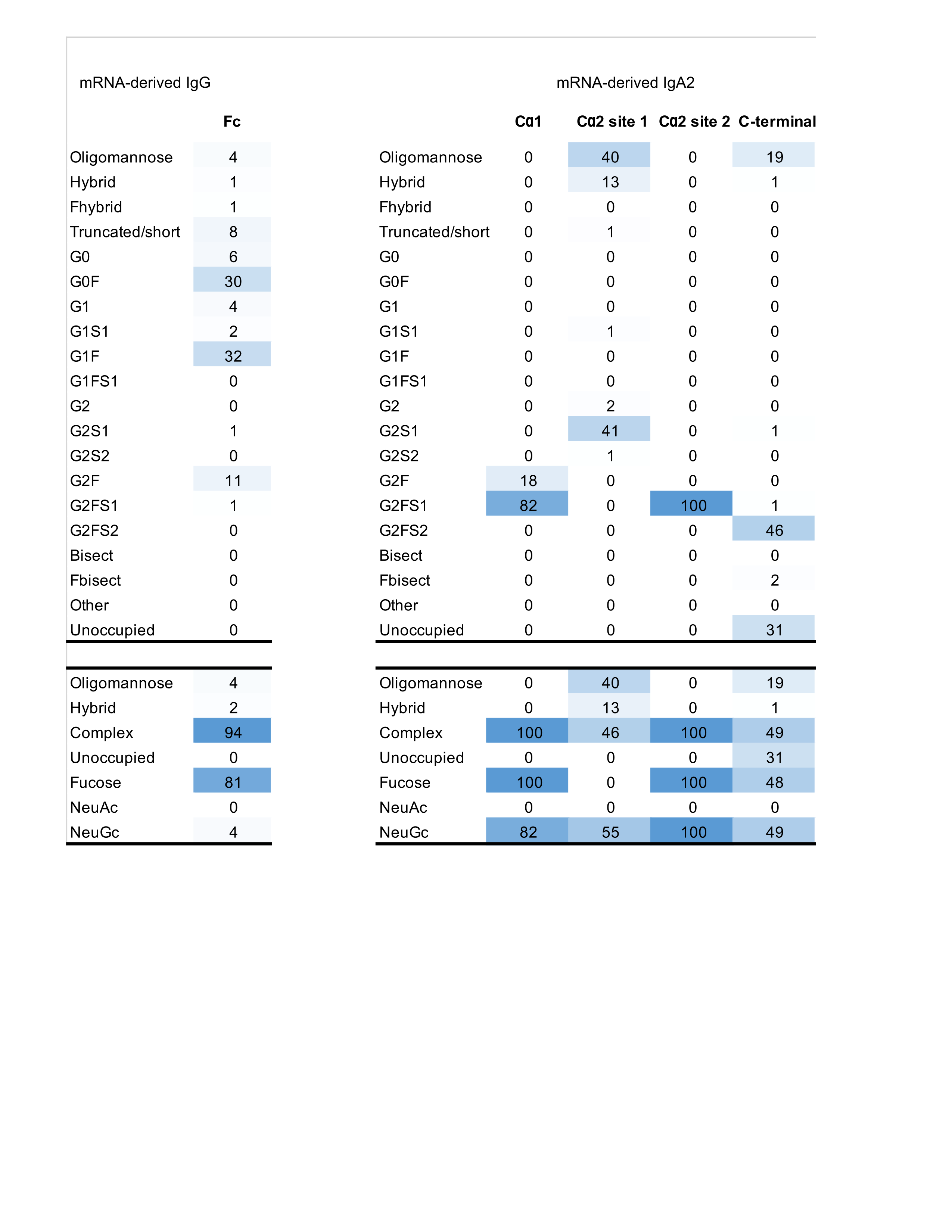

Supplement: Supplementary Table 5 — Site-specific glycosylation of IgG and IgA. [file Image8.tiff]
